# Exploring the adult sexual wellbeing and behavior during the COVID-19 pandemic. A systematic review and meta-analysis

**DOI:** 10.3389/fpsyt.2022.949077

**Published:** 2022-08-18

**Authors:** Iraklis Mourikis, Ioulia Kokka, Elli Koumantarou-Malisiova, Konstantinos Kontoangelos, George Konstantakopoulos, Charalabos Papageorgiou

**Affiliations:** ^1^First Department of Psychiatry, National and Kapodistrian University of Athens Medical School, Outpatient Specialty Clinic for Sexual Health Eginition Hospital, Athens, Greece; ^2^First Department of Psychiatry, National and Kapodistrian University of Athens Medical School, Eginition Hospital, Athens, Greece; ^3^Research Department of Clinical, Educational and Health Psychology, University College London, London, United Kingdom; ^4^Neurosciences and Precision Medicine Research Institute “Costas Stefanis” (UMHRI), University Mental Health, Athens, Greece

**Keywords:** COVID-19 restrictions, sexual satisfaction, sexual function, sexual frequency, sexual behavior, pandemic outcomes

## Abstract

Implemented social distancing measures may have forestalled the spread of COVID-19, yet they suppressed the natural human need for contact. The aim of this systematic review was to explore the impact of the COVID-19 pandemic on adult sexual wellbeing and sexual behavior. An extensive search in Pubmed, Scopus, and PsycInfo databases based on PRISMA guidelines was conducted. After applying specific eligibility criteria, screening resulted in 38 studies. Results were drawn from 31,911 subjects and outlined the negative effect of the pandemic in sexual frequency, function, satisfaction, and the behavioral changes regarding masturbation and internet-based practices. Meta-analyses of the drawn data on 1,343 female, and 1,372 male subjects quantified the degree of sexual function change during the COVID-19 pandemic vs. prior the pandemic. A random effects model revealed the significant negative impact of the pandemic on female sexual function (SMD: 0.76, 95% CI:0.74 to 1.59), while no significant change was found for the males (SMD: 0.25, 95% Cl: −0.03 to 0.52). Significant heterogeneity was identified across included studies (*p* < 0.00001, I^2^ = 97%, I^2^ = 90% for females and males, respectively). As part of the global health, sexual wellbeing should be on the focus of clinicians and researchers.

## Introduction

In response to the exponential growth in the COVID-19 infections’ number, nations worldwide implemented lockdowns and extensive -strict or more lose- measures, which had short- and long-term effects on health systems, education, economy, and several other societal segments (1–4). The restrictions were implemented with a solid purpose to mitigate the spread of the virus, yet they suppressed the natural human need for contact, and seem to have taken a toll on people’s mental wellbeing. Scientific evidence so far suggest that social distancing during the pandemic has led to higher levels of stress, and agitation (5). Leveraging data from studies in the midst of the restrictions around the globe reported elevated irritability and mood swings (6), and increases in both depressive and anxiety disorders (7), findings supported by meta-analytic reports (8).

The pandemic of COVID-19 could be perceived as a new type of trauma. Even though it does not fall into any of the Post Traumatic Stress Disorder (PTSD) models, the global scale of this stressor and the likelihood of this virus to become life threatening may result in similar symptomatology (9). For example, a large cross-national study highlighted that individuals who were infected or were afraid of getting infected demonstrated PTSD-like symptoms, introducing a “pathogenetic event memory model of traumatic stress” (10). Research has outlined that mental and sexual health undoubtfully share a strong, bidirectional link (9, 10). A large number of psychiatric anxiety-related entities demonstrate symptomatology which affect sexual wellbeing. The adverse relationship of anxiety and sexual gratification has been well documented (11, 12), as these indicators are inextricably linked to sexual desire, arousal, and satisfaction (13). Under chronic stressful circumstances, even though both males and females demonstrate increased sexual desire probably as a means of psychological relief, stressors prevent the progression of desire to actual sexual intercourse (14), resulting in reduced sexually physical contacts (15, 16). Complementary, international health associations such as WHO and CDC have highlighted the positive impact of sexual wellbeing on mental health. A healthy sexual life may function as a protective factor against psychopathology (17), while frequent sexual activity acts as a safeguard toward psychological wellness (18).

In the context of psychologically burdening feelings during the pandemic, physical intimacy -which could be considered as one of the core expressions of connection between romantic partners- could not have stayed intact, and alterations on people’s sexual relationships during the pandemic were expected. Nevertheless, given the fact that each sexual act is a multi-sensory experience that can take multiple forms, body contact is not always mandatory. Thereby, the question whether the COVID-19 pandemic has affected or altered the sexual wellbeing and behavior of individuals arises.

Researchers from various countries have tried to shed light on the impact of the pandemic on sexual health, and preliminary results have shown its influence on various aspects of sexual wellbeing (18, 19). However, drawing a clear conclusion based on the studies of the field could be misleading due to the diversity of the recruited samples. A few efforts to systematically approach findings on the matter have been attempted. To the authors’ knowledge, these were limited and relevant to safe sexual practices regarding transmission of the virus (20), sexual minorities (21), addressed only female subjects (22), or evaluated solely sexual function (23). Thus, the primary aim of this review was to systematically explore the potential impact of COVID-19 pandemic on aspects of sexual wellbeing, quantify the change with respect to sexual function, and identify probable behavioral changes.

## Materials and methods

The study was designed according the PRISMA statement guidelines (24), in order to identify papers relevant to sexual wellbeing and sexual behavior during the pandemic. Stages of research incorporated problem formulation, thorough search of the existing research in the field, data extraction and evaluation, and finally data analysis and presentation. Studies included in this review followed specific inclusion/exclusion criteria as indicated below. Sexual wellbeing is a broad construct, which lacks a sharp definition, expanding from sexual self-esteem to sexual experiences (25). For the purpose of this study, wellbeing is conceptualized as including pillars of sexual intimacy such as frequency, desire, function, and satisfaction.

### Eligibility criteria

For a study to be eligible, it had to evaluate relevant to sexual wellbeing aspects (e.g., sexual function/dysfunction, activity, satisfaction etc.) and/or sexual behaviors. The study had to involve adult-only subjects, regardless of gender, age, sexual orientation, and relationship/marital status. Study groups had to derive from the general population but not on subjects with sexual dysfunction established prior the pandemic. The studies had to be published in the English language by peer-reviewed journals. Studies including females during pregnancy or post-partum were excluded, as these states have been proven to affect sexual function in a negative fashion (26, 27). Studies that included subjects with mental illnesses were excluded, because of the effect specific psychotropic medication can have on sexual function (28). Studies that investigated the biological impact of the virus on sexual function of COVID-19 survivors were also excluded. Research protocols without providing sufficient data were not included as well.

### Search strategy

xPubmed, Scopus, and PsycInfo databases were thoroughly searched for relevant studies from the 1st until the 28th of March 2022. Research was conducted by two reviewing investigators, using the following terms: “*sexual function”* OR *“sexual dysfunction”* OR *“sexual activity”* OR *“sexual health”* OR *“sexual satisfaction”* and “*sexual behavior*” OR “*sexual practices*” OR “*sexual habits*” AND *“COVID-19”* OR *“coronavirus 2019”* OR *“lockdown”* OR *“pandemic”* OR “*quarantine”* and were adopted accordingly when necessary. Titles, keywords, and abstracts of each study were screened for eligibility. A backward search (hand search of reference lists) of included papers was conducted to identify additional studies relevant to the topic. All yielded studies were assessed according to the eligibility criteria.

### Data extraction and quality evaluation

Data extraction included country of origin, time point of the pandemic during which the study was held, the sample size of each study, participants’ mean age and gender, the aspect of sexual wellbeing under investigation, the measurements used, the main outcomes of individual studies, and any other piece of information required for the quality evaluation. The AXIS Appraisal Tool was used to assess each study’s quality (29). AXIS consists of 20 items with each measuring a different aspect of a study’s quality. The aim of the tool is to assist systematic interpretation of observational studies. Each question of the tool can be answered with “yes,” “no” or “do not know,” yet it is not used to generate a total quality score, due to the well-known problems associated with such scores (30). The procedure of data extraction was held by two reviewers. The quality of evidence was assessed with the use of the Grade of Recommendation, Assessment, Development, and Evaluation criteria (GRADE) (31). The criteria that were assessed for each study was sampling issues, consistency of methods and findings and precision of data curation.

### Quantitative synthesis and meta-analysis

A quantitative synthesis of findings regarding sexual function was performed for the studies that provided adequate information. The difference between established indices of sexual function (e.g., International Index of Erectile Function, Female Sexual Function Index etc.) before and during the pandemic was calculated using the standardized mean difference (SMD) with a 95% confidence interval (CI). The *Z*-test was used to determine the significance of the pooled SMD. The tau^2^ statistic was used to examine the standard deviation of underlying effects across studies. A random-effect model was applied after calculating Cochran’s Q-statistic (*p* < 0.05) and I^2^ test. A visual examination of the funnel plots was performed to estimate the publication bias. The statistical significance level was set at 5% (*p* < 0.05). Statistical analysis was performed using the Review Manager software (Version 5.4, the Nordic Cochrane Centre, Copenhagen, Denmark).

## Results

### Flow of the included studies

**FIGURE 1:**
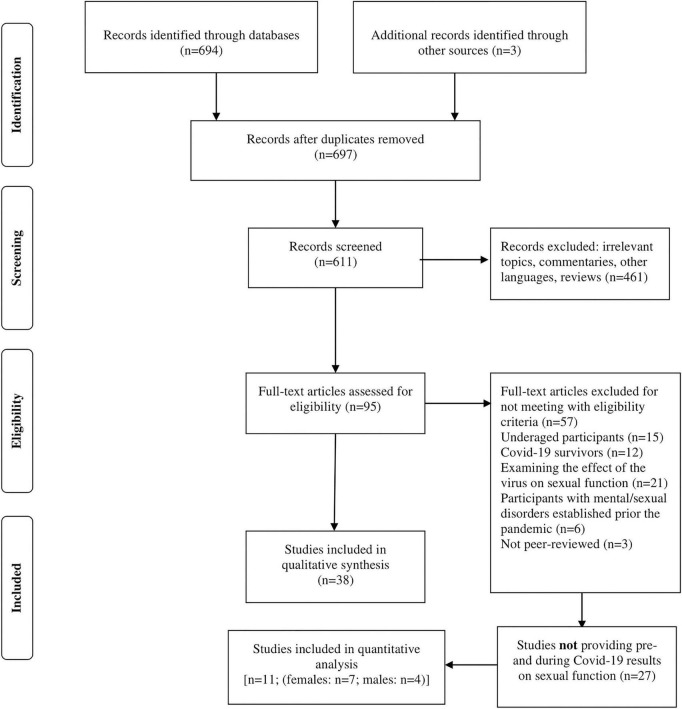
Flow diagram of the study selection of the included studies in the systematic review.

The initial search yielded 694 studies. After removing duplicates, and 611 titles and abstracts were screened, 95 articles were fully assessed. 57 of them were excluded for not meeting with the eligibility criteria. The final step of research resulted in 38 studies. Detailed screening procedure is illustrated in Figure 1.

### Main characteristics of the included studies

All studies were observational, and more specifically of cross-sectional design. The majority was conducted in Europe (*n* = 23) and during the first semester of the pandemic (*n* = 33), with 31,911 recruited subjects in total. Mean age of the participants was 34.5 years for 32 of the studies; six of them provided only the lower age limit for participation (>18 years old). Mean percentage of female participants was 64.6% among 20 studies. Nine of them examined solely female populations, six of them solely males, while one study did not clarify participants’ gender distribution. Five of the studies included exclusively coupled (married/cohabiting/non-cohabiting) participants. Four and six studies included exclusively homosexual and heterosexual participants, respectively. Five of the studies included participants differentiating their gender identity from the dyadic system (woman/man). Apart from the instruments and questionnaires used to evaluate sexual wellbeing, almost half of the included studies used tools to assess the mental state or wellbeing of their participants (*n* = 18). Thirteen studies used a combination of weighted questionnaires and structured interviews, 13 used solely weighted questionnaires, and 12 solely structured inquiries. Main characteristics of included studies are outlined in Table 1. Among the included studies frequency of sexual intercourse, general sexual satisfaction, sexual function, and specific sexual behaviors were examined.

**TABLE 1:** Characteristics of the studies included in this systematic review.

Authors, country, year of publication	Time points of study conduction	Sample’s characteristics [size;gender; relationship status; sexual orientation]	Sample’s age (mean age/SD, where applicable)	Aspect of sexual wellbeing/behavior under investigation	Instruments for outcomes of interest	Main results
Cocci et al., (60) Italy	February – April 2020	*n* = 1515; N/A	21.0/NA	Sexual well-being during COVID-19	Questions on sexual habits pre- and post quarantine, BDI-II, BAI	Significant decrease of sexual satisfaction, >50% reported complete absence of sexual satisfaction, lower age and higher BDI scores were significant predictors of sexual dissatisfaction for both genders. Almost 40% reported increase in masturbation
Fuchs et al., (45) Poland	March-April 2020	*n* = 764; 100% females; 68% non-cohabiting relationship, 24.8% married, 7.2% single; N/A	25.1 ± 4.3	Female sexual function and anxiety/stress related to COVID-19	FSFI, structured questionnaire on stress and anxiety	Statistically significant decrease in all subscales of the instrument (*p* < 0.001), decrease of sexual intercourse number, increased stress and anxiety levels.
Yuksel and Ozgor, (42) Turkey	March-April 2020	*n* = 58; 100% females; N/A	27.6 ± 4.4	Female sexual behavior during COVID-19	FSFI, menstrual status, frequency of sexual intercourse	Significantly increased sexual intercourse, better FSFI total score, and three domain scores for arousal, orgasm, and satisfaction were significantly higher prior to the pandemic.
Cito et al., (34) Italy	April-May 2020	*n* = 1576; 64.6% females;35.4% males; 96.8% in a steady relationship; N/A	38.0/NA	Couples’ sexuality changes during COVID-19 quarantine	Adapted scale on well-being, questions on sexual health domains and intercourse and autoerotism	Significant decline in well-being, correlation of well-being with Sexual Intercourse (SI), decreased SI, relation between reduced salary and SI, reasons for reduced SI was poor privacy and lack of psychological stimuli.
Lehmiller et al., (39) United States	March-April 2020	*n* = 1559; 71.1% females;23.4% males 4.5% non-binary; 52.7% heterosexuals; 7% homosexuals; 20.8% pansexual/bisexual/other identities	>18.0/NA	Changes in Sexual Behavior during the COVID-19	4-item PSS, UCLA loneliness scale-Revised, FSFI, questions on sexual changes during the pandemic, 49-item checklist on new sexual behaviors	43.5% reported a decline in the sexual life. Decreased sexual behaviors, 20.3% reported a new addition of sexual behaviors from the provided checklist.
Schiavi et al., (40) Italy	February-March 2020	*n* = 89; 100% females; N/A	39.0/NA	Female sexual function during lockdown	FSFI, FSDS, SF-36	Participants reported significant decrease in FSFI, and significant increase of FSDS scores post quarantine.
Arafat et al., (49) Bangladesh, India, Nepal	April 2020	*n* = 120; 77.5% males;21.7% males; 0.8% unidentified;100% married	35.42 ± 5.73	Sexual behavior of married couples during lockdown	structured questionnaire on sexual life	45% of the respondents reported that lockdown had some effect on their sexual intercourse number. 50% reported a positive effect on their emotional bonding with their spouse
Ilgen et al., (53) Turkey,	January-February 2020	n = 52; 100% females;100%stable relationship; 100%heterosexual	35.1 ± 5.8	Female sexual function during COVID-19 pandemic	FSFI, BDI, BAI	FSFI scores of the participants were higher before the pandemic, however, this finding was not statistically significant. BAI scores had a negative correlation with FSFI scores.
Bhambhvani et al., (50) United States,	March 2020	*n* = 91; 100% females; 82.4% stable relationship; 15.4% single; N/A	43.1 ± 11.8	Impact of the COVID-19 pandemic on female sexual function and frequency	FSFI, PHQ-4	Statistically significant decrease in FSFI total scores pre- and during- the pandemic. No significant change in sexual frequency was reported by most of the participants.
Sotiropoulou et al., (54) Greece,	April-May 2020	*n* = 299; 71.2% females; 29.8% males; 100% stable relationship;100% heterosexual	43.2/NA	Sexual function and relationship quality of heterosexual couples during the quarantine	FSFI, IIEF, structured questionnaire on sexual activity, relationship quality, and mood and anxiety	No statistically significant difference of FSFI scores pre- and during the pandemic. IIEF was statistically higher during the pandemic. Weak associations between depressive mood and anxiety and sexual well-being were reported.
Karagoz et al., (55) Turkey,	May 2020	*n* = 245; 39.6% females;100% stable relationship; 100% heterosexual	35.9 ± 6.9	The effect of COVID-19 pandemic on couples’ sexuality	GAD-7, PHQ-9, PSS, FSFI, IIEF	Thoughts for contraction during sexual intercourse were expressed (*p* = 0.002). Increased masturbation (*p* = 0.022). Significant decrease in the erectile and orgasmic function, intercourse satisfaction, and overall satisfaction scores (*p* = 0.001, *p* = 0.014, *p* = 0.001, *p* = 0.001, respectively). Statistically significant decrease in lubrication, orgasm, and satisfaction in women (*p* = 0.034, *p* = 0.023, *p* = 0.007).
Carvalho et al., (56) Portugal,	March-June 2020	*n* = 662; 62.9% females; 37.1% males;100% heterosexual; 61.6% cohabiting partners; 38.4% single	38.0 ± 12.0	Examination of the relationship between COVID-19 confinement and sexual functioning domains in heterosexual males and females	Self-reported levels of confinement and psychological adjustment during lockdown, IIEF, FSFI	Psychological adjustment mediated the effects of confinement in male sexual desire, erectile function, sexual satisfaction, and overall satisfaction; no mediating effects were found regarding orgasmic function. No significant correlation of confinement and female sexual function. Increased psychopathological symptomatology predicted lower levels of sexual desire, lubrication, arousal, satisfaction, and orgasm.
Karsiyakali et al., (37) Turkey,	June 2020	*n* = 1356; 50.5% females;49% males; N/A 47.8% married; 52.2% single; N/A	33.1 ± 8.31	The effects of the COVID-19 pandemic on the sexual functioning	IIEF, FSFI, questionnaire on sexual desire, masturbation and number of intercourses	Statistically significant decrease in sexual desire, masturbation and number of sexual intercourses. Being single, not having a child, having a regular sexual partner, and being unemployed were associated with a decline in sexual intercourse frequency and sexual desire.
Wignall et al., (58) United Kingdom,	May 2020	*n* = 467; 59.8% females; 60.4% stable relationship; 32.6% single; 7% causal relationship;86% heterosexual; 14% homosexual	25.3 ± 4.13	Changes in Sexual Desire and Behaviors during lockdown	SDI-2, sexual behavior catalogue, SOI-R	Significantly decrease in sexual desire for females, insignificant decrease for males. Sexual behaviors reduced during the pandemic, 20% reported increased use of pornography. 33% reported having less sex, and 25% masturbating less. Men and LGB individuals reported greater increases in sexual activity than women and heterosexuals.
Panzeri et al., (48) Italy,	April-May 2020	*n* = 124; 73.4% females;26.6% males;94.4% heterosexual; 4% bisexual;1.6% homosexual	34.01 ± 8.71	Changes in sexuality and quality of couple relationship during the COVID-19 lockdown	BISF-W, SDI, DASS-21, PHQ-15, QMI	No changes in sexual desire, arousal, and orgasm during lockdown for males and females. 24.2% of the males and 30.8% of the females reported a decrease in sexual frequency
Luetke et al., (38) United States,	April 2020	*n* = 742; 51.0% females; 49% males; 81% stable relationship; 19% single; N/A	44.0/NA	Changes in intimate and sexual behaviors and experiences during COVID-19	UCLA Loneliness scale, CES-D-10, questions on sexual behaviors and frequency, and orgasm and emotional closeness	Frequent coronavirus-related conflict was significantly predictive of decreased frequency of solo and partnered intimate and sexual behaviors.
Hille et al., (36) Germany, Switzerland and Austria,	April 2020	*n* = 2515; 47.4% females; 53.6% males; 77.6%; N/A; heterosexual;13.2% bisexual; 6.7% homosexual; 2.4%pansexual	44.0/N/A	Changes in sexual behavior during the COVID-19 pandemic	Questionnaire on sexual activities and practices, personal satisfaction	Significant decline in frequency of sexual activities since the distancing measures. Only anal intercourse showed no significant decrease. Those in a relationship masturbated significantly less during the pandemic.
Baran and Aykac, (33) Turkey,	June 2020	*n* = 536, 100% males; 75.4% stable relationship; 24.6% single; N/A	38.6 ± 10.3	Effect of COVID-19 fear on sexual behavior	IIEF, questions on fear of transmission and changes in sexual behavior	19.4% (104) developed fear of COVID-19 transmission from the sexual partner. Statistically significant decrease of weekly sexual intercourse
Cascalheira et al., (65) United Kingdom,	May 2020	*n* = 565, 59.8% females; 38.9% males; 0.9% non-binary; 86.1% heterosexual; 9.2% bisexual; 4.6% homosexual	25.4 ± 4.1	Changes in Sexual Fantasy and Solitary Sexual Practice	Questions on solitary sexual behaviors, sexual fantasies and pornography consumption	34.3% engaged in more sexual fantasizing, 30.44% reported an increase in solitary sexual practice, increase in pornography use for 19%
Gouvernet and Bonierbale, (62) France,	April-May 2020	*n* = 1079; 68.7% females; 31.3% males; 20.7% single; 79.3% stable relationship; N/A	>18.0/NA	Impact of COVID-19 on sexual cognitions and emotions	SMQ, GAD7, MDI, ECR-RS, questions on sexual frequency and satisfaction	Decrease in sexual frequency and satisfaction, which affected mostly women, and were related to negative sexual cognitions and less positive sexual emotions. Increases in digital sex use contributed to minimizing the likelihood of negative sexual motions
Hammoud et al., (46) Australia,	April 2020	*n* = 940, 100% males; N/A; 92.7% homosexual; 7.3% bisexual	39.9 ± 13.4	Disrupted Sexual Behaviors Among Gay and Bisexual Men	Questions to measure changes in sexual behaviors	84.2% reduction is sexual intercourse during the pandemic compared to before the outbreak
Osur et al., (64) Kenya,	September 2020	*n* = 194, 39.2% females, 60.8% males; 100% married; heterosexual	>18.0/NA	Perceived and experienced sexual satisfaction among married couples during COVID-19	Questionnaire adapted from the Index of Sexual Satisfaction	41.3% reported being sexually dissatisfied, 26.6% reported being dissatisfied prior to the pandemic. Significant difference when comparing before and during COVID-19 sex satisfaction (χ^2^ = 38.86, *p* < 0.001).
Mumm et al., (52) Germany,	April-July 2020	*n* = 414, 100% cis males; 62.1% stable relationship; 37.9% single; N/A Hetero- Homo- and Bisexual	>18.0/NA	Sexual Behavior of Hetero-, Homo-, and Bisexual Males	Sexual Behavior Questionnaire	Average weekly frequency of sexual intercourse and masturbation was increased in all groups, significant rise satisfaction with the sexual frequency, level of sexual arousal increased significantly in all groups, joy from sexual intercourse or masturbation increased significantly in heterosexual (*P* < 0.0005) and homosexual men (*P* < 0.005)
McKay et al., (67) United States,	April-May 2020	*n* = 728, 100% males; 53.7% stable relationship; 46.3% single; N/A homosexual, bisexual	>18.0/NA	Sexual Behavior Change Among Gay and Bisexual Men	Questions on sexual behavior	9 out of 10 participants reported having sex with a stable partner or no sex at all. Reporting no sexual partners in the last 30 days was significantly predicted by increased exposure to a Stay-at-Home order. Increased masturbation and cyber-sex. HIV-positive men were particularly likely to adopt strategies including avoiding casual partners
López-Bueno et al., (51) Spain,	March-May 2020	*n* = 536, 72.8% females; 27.2% males; 33.2% stable relationship; 66.8% single; N/A	>18.0/NA	COVID-19 Confinement and Sexual Activity in Spain	Questions on sexual activity	No significant difference in sexual activity was reported, particularly for those married or in a domestic partnership.
Gasso et al., (68) Spain,	March-June 2020	*n* = 293, 66.2% females; 32.8% males; 1% unspecified; 83.6% heterosexual; 5.1% homosexual; 10.2% bisexual; 41% single; 59% stable relationship	30.3 ± 13.0	The prevalence of sexting and online sexual victimization behaviors	Sexting items adapted from the Juvenile Online Victimization Questionnaire	Sexting engagement and online sexual victimization decreased during lockdown despite the increase in internet use
Shilo and Mor, (66) Israel,	March-April 2020	*n* = 2562; 100%males; 100% homosexual; 81.6% single; 18.4% stable relationship	37.0 ± 11.3	Changes in sexual behavior of MSM during the COVID-19 pandemic	questions on sexual activity, practices, frequency and number of partners	39.5% continued to meet new casual sex partners. Being younger, single, and with higher mental distress predicted engagement in casual sex. MSM reduced their sexual risk and limited sexual repertoire
Neto et al., (63) Brazil,	July-August 2020	*n* = 1314; 70.6% females; 29.4% males; 89.2% heterosexual; 10.8% homosexual; 82.6% steady relationship; 17.4% single	37.6 ± 10.8	Impact of the pandemic on sexual function	FSQ, MSQ, questions on sexual behaviors and libido	Worsening of sexual satisfaction was reported by 44.5% of the participants, with the following associated factors: Lower libido, missing Nightlife, Higher Masturbatory Frequency, and isolation from the partner. Worsening of Libido was reported by 37%.
Costantini et al., (59) Italy,	May 2020	*n* = 2149, 51.7% females; 48.3% males; 94% heterosexual; 10% bisexual; 4% homosexual; 84.2% stable relationship; 15.8% single	43.0 ± 12.5	Changes in the sexual behavior of adult men and women in stable relationships	IIEF, FSFI, marital adjustment test, Hamilton Anxiety Rating Scale	The sex lives improved for 49% of participants, particularly those in cohabitation, for 29% it deteriorated, while for 22% of participants remained stable.
Ballester-Arnal et al., (43) Spain,	April 2020	*n* = 1478; 67.5% females; N/A; N/A	31.92 ± 10.1	Sexual habits of the general population during lockdown	Questions on sexual desire and activity, masturbation, sexual relationships, online sexual activity, sexual fantasies and urges	Confinement affected the sexual life of almost half of the sample (47.7%), mostly females. Those with a worsen sexual life were 3 times more (37.9%) than those who reported an improvement.
Coombe et al., (44) Australia,	April-May 2020	*n* = 965; 70.0% females; 25.6% males; 4.4% gender diverse; 61.8% stable relationship; 38.2% single; 65.7% heterosexual; 29.4% homosexual; 4.9% bisexual	24.0/NA	Impact of lockdown on sexual practices	Questions on trends and changes in sexual practices, intimate relationships	53.5% reported less sex during lockdown. Solo sex activities increased; 14.6% reported using sex toys more often and 26.0% reported masturbating more. Using dating apps for chatting/texting and setting up virtual dates increased during lockdown.
Ates et al., (32), Turkey	November-December 2020	*n* = 602, 100% males; 60.1% married; 39.9% single; N/A	36.1 ± 11.6	Heterosexual male changes in sexual function and behavior	IIEF, IELT, PEDT, sexual intercourse frequency	Statistically significant reduction of sexual frequency (*p* < 0.001), total IIEF score significantly lower (*p* < 0.001), subscales of sexual function and satisfaction were significantly higher (*p* = 0.016, *p* < 0.001 respectively). PEDT score significantly higher (*p* = 0.004). No significant difference in IELT.
Szuster et al., (41) Poland	April-May 2020	*n* = 1644, 100% females; 83.1% stable relationship; 16.9 single; N/A	25.11 ± 7.09	Impact of COVID-19 on mental and sexual health of reproductive aged women	FSFI, BDI	Lower frequency of sexual activity (*p* < 0.001) and a lower libido level (*p* < 0.001). Mean FSFI total score was 27.01 ± 7.61. SFI and BDI scores were significantly correlated (*P* < 0.001).
Gleason et al., (61) United States	October 2020	*n* = 1051, 57.3% males; 42.7% males; 65.5% stable relationship; 34.5% single; 88.3% heterosexual; 3.4% homosexual; 6.3% bisexual; 1.1% pansexual	38.54 ± 10.56	Impact of COVID-19 on sexual behaviors	Questions on sexual frequency, satisfaction and sexual/physical violence	Significant but small (*d* < 0.2) increase in masturbation and pornography use for males. Significant decrease in sexual desire for females. Small significant decreases (*d* > 0.2) sexual enjoyment/pleasure, and a medium significant decrease (*d* > 0.5) was noted for frequency of sex with casual partners
Grover et al., (35) India	May-June 2020	*n* = 450, 85.6% males; 14.4% females; 95.1% stable relationship; 4.8% single; N/A	41.5 ± 11.2	Sexual function during the pandemic	CSFQ, PHQ-4	Statistically significant reduction in sexual frequency (*p* < < 0.001) and intimate contact when not involved in sexual practices, e.g., hugging/cuddling (*p* = 0.042).
Caruso et al., (69) Italy	Not provided*	*n* = 317, 100% females; N/A; N/A	18-48	Sexual activity and contraception use during the pandemic	Structured inquiry regarding contraception and sexual activity	All married and cohabiting women were continuing to use their contraceptive method. 50.5% non-cohabiting or single women had discontinued their contraception method while social distancing, for non-method-related reasons. 46.5% non-cohabiting or single women had continued their sexual activity, infringing social distancing rules, and 14.9% had had an unplanned pregnancy, for which they had sought a termination.
Kusuma et al., (47) Indonesia	November-December 2020	*n* = 131, 48.9% females; 51.1% males; 71.9% married; 28.1% single; 96.1% heterosexual; 2.2% homosexual; 1.5% bisexual	28.7/N/A	Differences in mood and sexual activity during COVID-19	DISC, questions on behavior, and frequency of sexual intercourse before and during COVID-19 pandemic	53.8% of respondents admitted that the COVID-19 pandemic affected their sexual activity. No significant difference in condom use between before and after the pandemic was noted.
Chatterjee et al., (57) India	July-August 2020	*N* = 1376, 80.5% males; 19.4% females; 65.9% married; 34.1% single; N/A	34.42 ± 9.34	Association between sexual function and mental comorbidities and quality of life during the pandemic	DASS21, ASEX, WHOQOL-BREF	27.18% reported having a sexual dysfunction based on the ASEX instrument. Increase in age and female gender were associated with sexual dysfunction overall and also all its components. Increased depressive symptomatology was associated with lack of orgasm, and sexual satisfaction.

SD, Standard Deviation; FSFI, Female Sexual Function Index; PSS, Perceived Stress Scale; BDI-II, Beck’s Depression Inventory-II; BAI, Beck’s Anxiety Inventory; STAI, State-Trait Anxiety Inventory; CES-D-10, Center of Epidemiologic Studies Depression Scale, SF-36, 36 Short Form Health Survey, SF-36, 36 Short Form Health Survey, GAD-7, Generalized Anxiety Disorder-7, PHQ-9, Patient Health Questionnaire-9, ISS, Index of Sexual Satisfaction; SDI-2/SDI, Sexual desire inventory; SOI-R, Sociosexual orientation inventory-Revised; ECR-RS, Experiences in Close Partner Attachment Scale; MDI, Major Depression Inventory; SMQ, Sexual Mode Questionnaire, FSQ, Female sexual quotient; MSQ, Male sexual quotient, BISF W/M, Brief Index of Sexual Functioning (Women/Men); QMI, Quality of Marriage Index; FSDS, Female Sexual Distress Scale; MSM, males having sex with males; IELT, Intravaginal ejaculatory latency time; PEDT, Premature ejaculation diagnostic tool; CSFQ, Changes in Sexual Functioning Questionnaire; PHQ-4, Patient Health Questionnaire-4; DISC, Depression Intensity Scale Circles, DASS21, Depression Anxiety Stress Scale 21; ASEX, Arizona Sexual Experience Scale; WHOQOL-BREEF, WHO quality of life questionnaire; *, Authors have tried to contact the research team in order to find the time frame of the study without success.

### Main findings regarding the research questions

#### Frequency of sexual activity

The domain of sexual frequency was examined by a large portion of the included studies (*n* = 21). Participants were asked to report their sexual frequency on a weekly basis compared to the period prior the pandemic. In the majority of the studies sexual frequency referred exclusively to partnered sexual practices (mutual masturbation, vaginal/anal penetration etc.), while in one study masturbatory or other solo activities were examined. Eleven of them found a statistically significant decrease in the number of sexual interactions during the pandemic (32–42). Notably, one study reported that about 60% of their participants did not engage in any form of partnered sexual intercourse since the outbreak of COVID-19 (39). Six of the included studies reported a decrease in the frequency of sexual activity (39, 43–48), and the proportions of participants reporting decrease ranged from 14 to 53%. However, the reduction in these studies was not statistically significant. For two of the studies no change was found (49, 50), while one, which examined the frequency of both partnered and solo practices (e.g., masturbation), reported increased frequency of sexual activity (51). Only one study found statistically significant increase in partnered sexual activity, with almost 30% being sexually active more than three times per week (52).

#### Sexual function

Sexual function and potential indications of sexual dysfunction were explored by 14 of the included studies. The majority of these studies (*n* = 12) examined this domain with the Female Sexual Function Index and the International Index of Male Function while the remaining with questionnaires structured by the researchers, and results could be considered contradicting. Eight included solely female participants, seven of which compared their results with pre-COVID data. Among them two reported statistically significant decreases in all domains of sexual function (desire, arousal, lubrication, arousal, satisfaction, and pain) (40, 45), and one came into similar decreases, yet those were statistically insignificant (53). Two of them found significant decreases for the global sexual function, but when subdomains were accessed separately, they concluded in statistical decrease only for arousal, lubrication, and satisfaction (42, 50). In two studies sexual function was evaluated for both male and female participants and no significant change was found for either of their subgroups (48, 54). In contrast, another study reported significant decreases for their female participants but only for the lubrication, orgasm, and satisfaction subdomains of sexual function, whereas significant decreases in global function and erectile and orgasmic function, and satisfaction sub-domains were found for their male participants (55). Two of the studies found that lowered sexual function was present only when the psychological burden of the restrictions was assessed as high (56), and mostly for females and older participants (57). Three of the included studies evaluated solely sexual desire. One found decrease for males and females but this was significant only for females (58), while the second one reported significant decrease for both sexes (37). Though Cito et al. reported a decreased number of sexual intercourses, they found stable and in a subset of the subjects increased levels of sexual desire (34). Ates et al found a significant reduction in IIEF total scores, but a significant increase for the subscale of sexual function, and significant increase in the premature ejaculation diagnostic tool (32).

#### General sexual satisfaction

General satisfaction deriving from the sexual life of individuals was examined by 15 of the included studies. One of the studies concluded in improved levels of satisfaction for more than 50% of their participants (49), while in one stability (22%) and improvement (49%) was found (59). Two of the studies found that only a small portion of their participants reported decreased sexual satisfaction and, akin to other studies, this occurred only in those demonstrating high levels of anxiety (53, 56). Five of them found a statistically significant decrease, while for one of them this was more prominent for the female participants (34, 41, 60–62). Interestingly, in one of them 50% reported complete absence of satisfaction (60). Among four of the studies, the reported deteriorated satisfaction ranged from 41.3 to 50% (39, 43, 63, 64), while experiencing fear and anxiety for contracting the virus and increased depressive symptomatology was significantly associated with lower sexual satisfaction (33, 41). Mumm et al who included solely males, resulted in increased sexual satisfaction but only for those of hetero- and homosexual orientation, and not for bisexual men (52).

#### Sexual behavioral changes

Seventeen of the included studies attempted to report on possible behavioral changes with respect to sexual life, such as masturbation frequency, online activities etc. Masturbation was examined by seven studies. All found a significant increase of masturbation (39, 43, 52, 55, 61), and the percentages of this increase ranged from almost 15 (44) to 40% (60) of the participants. On the contrary, three of the studies reported the exact opposite; a decrease in masturbation was found (58), however this was significant only for participants in stable and cohabiting relationships (36, 37). Digital and internet-based sexual practices were examined by a portion of the studies (*n* = 6). Three of them found an increase in pornography use, and this was reported by a similar percentage of participants (≈20%) across all studies (58, 65, 66). A 33% increase in cybersex was reported by one of the studies (67), while one found that almost 30% of their participants created and shared sexual, digital content for the first time (68), and one reported an increase in dating applications use and virtual dating (44). Changes in the sexual repertoire were additionally examined. Cascalheira et al found that more than 30% of their participants expanded their solo sexual practices such as fantasizing more (65), while Lehmiller et al reported additions in their participants’ sex lives, which included new positions during intercourse (1/5 of the participants), sharing (13%) and acting (8.5%) on fantasies, and using sex toys (7.3%) (39). Sexual positions were examined by one more study, which found statically significant decrease in face-to-face positions in order to avoid transmission of the virus (32). Behaviors of intimacy were similarly deteriorated, with two studies reporting significant reduction of romantic practices such as hugging or cuddling (35, 36). Two of the included studies examined the use of contraceptive measures, and results were contradicting; one revealed a more than 50% decline for non-cohabiting partners (69), and one reported no change (47). Three studies, which all included males who have sex with males, concluded in contradicting results; two found increased masturbation and cybersex (67), and avoidance of casual sexual intercourse with this reaching 15times fold reduction (46), while Shilo and Mor reported that almost half of their participants continued casual intercourse, but with limited repertoire (66). A summary of the findings are reported in Table 2.

**TABLE 2:** Summary reporting on changes in main outcomes of interest.

Sexual variable	Studies (ref. no)	Change in Outcome
Frequency (*n* = 21)	(32–42)	Statistically significant decrease
	(39, 43–48)	Statistically insignificant decrease
	(49, 50)	No change
	(51)	Statistically insignificant increase
	(52)	Statistically significant increase
Satisfaction (*n* = 15)	(34, 41, 60–62)	Statistically significant decrease
	(33, 39, 43, 63, 64)	Statistically insignificant decrease
	(49, 52, 53, 56, 59)	Statistically insignificant stability/increase
Behavioral changes (*n* = 17)	(39, 43, 44, 52, 55, 60, 61)	Statistically significant increase in masturbation
	(36, 37, 58)	Statistically insignificant decrease in masturbation
	(44, 58, 65–68)	Statistically significant increase in internet-based sexual practices
	(32, 35, 36, 39, 47, 69)	Alteration/expansion of sexual repertoire
Function (*n* = 14)	(32, 36, 37, 40, 42, 45, 50, 55–58)	Statistically significant decrease
	(34, 48, 54)	No change
	(53)	Statistically significant increase

#### Factors affecting sexual wellbeing and behavior

Some of the included studies tried to identify factors which mediated the relationship between sexual wellbeing and the pandemic. Factors regarding sociodemographic characteristics, as well as psychological characteristics were found to affect this relationship. The most prominent characteristic was that of gender; women appeared as mostly affected in a negative manner (43, 58, 61, 62) Socio-economic status (39), and reduced salary due to work suspension (34), unemployment (37, 59), lack of privacy (43, 48), younger age (39, 60, 66), and being single (69) were identified among these factors. With respect to psychological characteristics, increased depressive symptomatology (48, 53, 60), anxiety (48, 54), stress (32, 48), and loneliness (41) were identified τ*o* negatively affect sexual wellbeing. In addition, fear of contracting the virus was found to act as a restrictive factor of sexual wellbeing (41).

### Quality assessment

Two independent reviewers assessed the quality of individual studies with the Appraisal Tool for Cross-Sectional Studies (AXIS). Each study’s quality was evaluated independently by each reviewer and juxtaposed their results; no disagreements occurred. Overall quality did not vary significantly across studies, with most of them being of moderate quality. The main quality issues were the lack of information on non-responders, and questionable internal consistency of several studies due to the use of not validated instruments. An additional quality issue regarding sampling that needs to be addressed is the fact that all of the studies recruited their samples online, questioning their representativeness. Detailed outcomes of the quality evaluation are presented in Table 3. The GRADE evaluation method uses a 4-level system of evidence grading, with randomized control trial being the only type of study design that can receive 4/4 (high level of evidence). Given that all included studies were observational, the highest possible grade was 3/4 (moderate level of evidence), unless there was a reason to upgrade or downgrade. The risk of bias was assessed by evaluating the representativeness of sampling, and the measurement and reporting bias. The vast majority of the studies downgraded to 2/4, given the unjustified samples’ size (*n* = 35), and the use of structured inquiries to evaluate outcomes of interest (*n* = 15).

**TABLE 3:** Quality assessment of individual studies included in the systematic review based on the AXIS tool.

STUDY																																						

ITEM	Cocci	Fuchs	Yuksel	Cito	Lehmiller	Schiavi	Arafat	Ilgen	Bhambhvani	Sotiropoulou	Karagoz	Carvalho	Karsiyakali	Wignall	Panzeri	Luetke	Hille	Baran	Cascalheira	Gouvernett	Hammoud	Osur	Mumm	McKay	Lopez-Bueno	Gasso	Shilo	Neto	Costantini	Ballester-Arnal	Coombe	Ates	Szuster	Gleason	Grevor	Caruso	Kusuma	Chateerjee
Clearly stated objectives	Y	Y	Y	Y	Y	Y	Y	Y	Y	Y	Y	Y	Y	Y	Y	Y	Y	Y	Y	Y	Y	Y	Y	Y	Y	Y	Y	Y	Y	Y	Y	Y	Y	Y	Y	Y	Y	Y
Appropriate study design	Y	Y	Y	Y	Y	Y	Y	Y	Y	Y	Y	Y	Y	Y	Y	Y	Y	Y	Y	Y	Y	Y	Y	Y	Y	Y	Y	Y	Y	Y	Y	Y	Y	Y	Y	Y	Y	Y
Population clearly defined	N	Y	Y	Y	Y	Y	Y	Y	Y	Y	Y	Y	Y	Y	Y	Y	Y	Y	Y	Y	Y	Y	Y	Y	Y	Y	Y	Y	Y	Y	N	Y	Y	Y	Y	Y	Y	Y
Representantive sample	Y	Y	Y	Y	Y	Y	Y	Y	Y	Y	Y	Y	Y	Y	Y	Y	Y	Y	Y	Y	Y	Y	Y	Y	Y	Y	Y	Y	Y	Y	Y	Y	Y	Y	Y	N	N	Y
Proper selection process	N	N	N	N	N	N	N	N	N	N	N	N	N	N	N	N	N	N	N	N	N	N	N	N	N	N	N	N	N	N	N	N	N	N	N	N	N	Y
Address non-responders	N	N	N	N	N	Y	N	N	N	Y	N	N	N	N	N	N	N	Y	N	N	Y	Y	N	N	N	N	Y	N	Y	Y	Y	N	Y	Y	Y	N	N	Y
Appropriate measures	D	Y	Y	Y	Y	Y	D	Y	Y	Y	Y	Y	Y	Y	Y	Y	N	Y	Y	Y	Y	Y	Y	Y	Y	Y	Y	Y	Y	Y	Y	Y	Y	Y	Y	D	D	Y
Reliable measures	Y	Y	Y	Y	Y	Y	Y	Y	Y	Y	Y	Y	Y	Y	Y	Y	Y	Y	D	Y	D	Y	D	D	D	Y	D	Y	Y	Y	Y	Y	Y	D	Y	D	D	Y
Determined stat. significance	Y	Y	Y	Y	Y	Y	Y	Y	Y	Y	Y	Y	Y	Y	Y	Y	Y	Y	Y	Y	Y	Y	Y	Y	Y	Y	Y	Y	Y	Y	Y	Y	Y	Y	Y	N	N	Y
Sufficient methods description	Y	Y	Y	Y	Y	Y	Y	Y	Y	Y	Y	Y	Y	Y	Y	Y	Y	Y	Y	Y	Y	Y	Y	Y	Y	Y	Y	Y	Y	Y	Y	Y	Y	Y	Y	Y	Y	Y
Data adequately described	Y	Y	Y	Y	Y	Y	Y	Y	Y	Y	Y	Y	Y	Y	Y	Y	Y	Y	Y	Y	Y	Y	Y	Y	Y	Y	Y	Y	Y	Y	Y	Y	Y	Y	Y	N	N	Y
Possibility of non-response bias	Y	D	D	Y	D	N	Y	D	D	N	D	D	D	D	D	D	Y	N	D	D	D	N	D	D	D	D	N	N	N	D	N	D	N	N	N	Y	Y	N
Non-responders information	Y	N	N	N	N	Y	N	N	N	Y	N	N	N	N	N	N	N	Y	N	N	N	Y	N	N	N	N	Y	N	Y	Y	N	N	Y	Y	Y	Y	N	Y
Results internally consistent	D	D	Y	D	D	Y	D	Y	Y	D	Y	Y	Y	Y	Y	D	Y	Y	D	D	D	Y	D	D	D	Y	D	Y	Y	D	D	Y	Y	D	Y	D	D	Y
Results based on methods	Y	Y	Y	Y	Y	Y	Y	Y	Y	Y	Y	Y	Y	Y	Y	Y	Y	Y	Y	Y	Y	Y	Y	Y	Y	Y	Y	Y	Y	Y	Y	Y	Y	Y	Y	Y	Y	Y
Results justify conclusions	Y	Y	Y	Y	Y	Y	Y	Y	Y	Y	Y	Y	Y	Y	Y	Y	Y	Y	Y	Y	Y	Y	Y	Y	Y	Y	Y	Y	Y	Y	Y	Y	Y	Y	Y	Y	Y	Y
Limitations	Y	Y	Y	Y	Y	Y	Y	Y	Y	Y	Y	Y	Y	Y	Y	Y	Y	Y	Y	Y	Y	Y	Y	Y	Y	Y	Y	Y	Y	Y	Y	Y	Y	Y	Y	Y	N	Y
Conflict of interest	Y	N	N	N	N	N	N	N	N	N	N	N	N	N	N	N	D	N	N	D	D	D	N	N	N	N	Y	N	N	N	N	N	N	N	N	N	N	Y
Ethics approval	D	Y	Y	D	Y	Y	D	Y	Y	Y	Y	Y	Y	Y	Y	Y	D	Y	D	D	N	Y	Y	Y	Y	Y	Y	Y	Y	Y	Y	Y	Y	Y	Y	Y	Y	Y

Y, Yes; N, No; D, Do not know.

### Quantitative synthesis and meta-analysis

**FIGURE 2:**
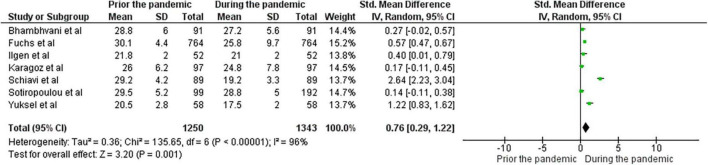
Forest plot presenting the meta-analysis based on SMDs for the effect of the pandemic on female sexual function.

**FIGURE 3:**
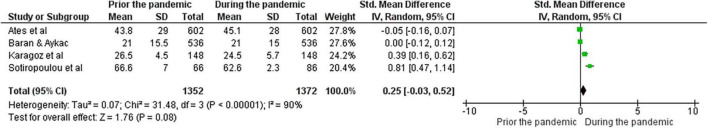
Forest plot presenting the meta-analysis based on SMDs for the effect of the pandemic on male sexual function.

**FIGURE 4:**
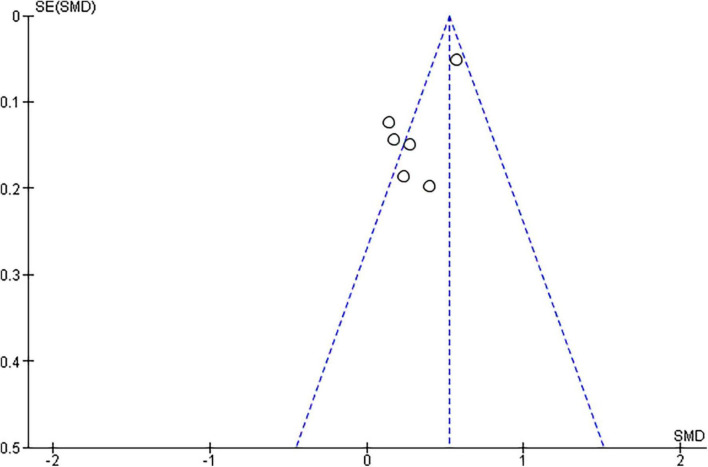
Funnel plots for the examination of publication bias for the females.

**FIGURE 5:**
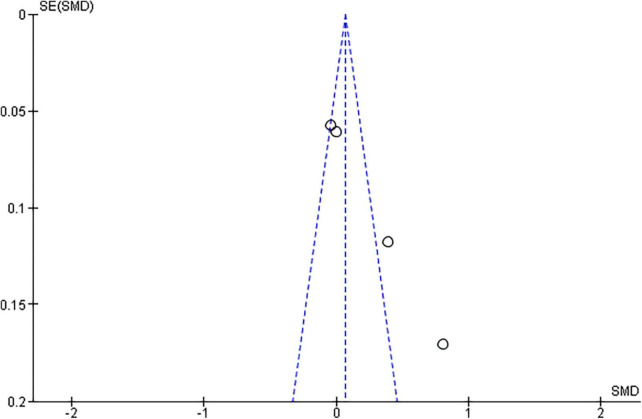
Funnel plots for the examination of publication bias for the males.

Among the included studies, only seven provided the required pre- and during-the pandemic data for their female participants, and four for their male participants. With respect to females, the random effects model revealed that the Female Sexual Function Index (FSFI) total scores showed a statistically significant difference between pre-COVID and during-COVID total scores for their participants (SMD: 0.76, 95% CI:0.74 to 1.59, z summary effect size *p* = 0.01) (Figure 2). Regarding males, the model showed that IIEF total scores demonstrated no significant difference between pre-COVID and during-COVID total scores for their participants (SMD: 0.25, 95% Cl: −0.03 to 0.52, z summary effect size *p* = 0.08) (Figure 3). A significant heterogeneity was identified across included studies (*p* < 0.00001, I^2^ = 97%, I^2^ = 90% for females and males, respectively). Visual examination of the funnel plots indicated the risk of publication bias over included studies (Figures 4, 5). Given the high heterogeneity of the studies, the authors intended to perform a meta-regression to investigate whether the results were influenced by other covariates. Due to the lack of adequate data, this was not feasible. However, among the covariates, the severity of the pandemic among different countries, the different type of restrictions implemented, or the different relationship status among the participants could be identified.

## Discussion

This systematic review aimed to examine the existing body of evidence regarding the influence of the COVID-19 pandemic on the sexual wellbeing and sexual behaviors of adults. Findings on the matter appeared to be rather consistent across studies, and partially supported by the meta-analytic outcomes. A deterioration of sexual wellbeing with lowered frequency of sexual activity, diminished satisfaction and, for some, problematic sexual function was found, whereas in only a small number of studies the pandemic was presented as an opportunity to reinvent intimate relationships. The relation of the participants’ psychological state and sexual wellbeing was evident. Sexual wellbeing and discomforting feelings, such as anxiety, increased stress, depressive symptomatology, and perceived lower quality of life were associated in a large portion of the included studies. However, the causal relationship could not be defined with certainty. As far as changes in sexual behavior are concerned, results were rather contradicting regarding masturbation, while a rise in internet-based sexual practices, and changes in sexual repertoire were documented.

Sexual function was the only aspect of sexual wellbeing for which data could be drawn to perform a meta-analysis. Results from the quantitative synthesis revealed a statistically significant negative effect of the pandemic on female sexual function. Taking into consideration that for most of the studies lower sexual function was linked to lowered quality of life and increased anxiety levels, this came in line with previous findings, which showed that chronic daily stressors can affect genital arousal and impair female sexual function (70). On the contrary, meta-analytic findings for the male sexual function showed no significant alteration. It may be supported that the meta-analytic findings outline the moderating role of gender, which emerged in several of this review’s studies (43, 48, 56, 58, 62). This could be partly explained by the fact that males are less susceptible to chronic stress (71). Because of different levels of exposure to psychological and social pressure, and increased vulnerability due to biological factors, women are more likely to be affected by stressful circumstances compared to men (72). However, it could be argued that the meta-analytic results for the male participants are not representative of the actual case. Among the included studies, those with the largest male samples demonstrated statistically significant reductions in sexual function, even though the comparison with pre-pandemic scores was not feasible. An additional argument could be that the IIEF index perceives male sexual function in a somehow narrow manner, since it examines solely penile rigidity and penetration, without assessing other ways males can engage in and enjoy sexual intercourse. Complimentary, a plausible explanation for the statistically insignificant findings could be the reported increase of medication regarding male sexual dysfunction. A recent study found that between February and December of 2020 a 67% increase in sales of phosphodiesterase-5 inhibitors (PDE5-Is) was noted in the United States, and more particularly, an 85% increase of tadalafil sales (73). This suggests that men’s function might not have been affected, but pharmaceutical assistance was required.

With respect to sexual frequency, the limited number of intercourses could be characterized as expected. Literature has shown that emotional and physical intimacy play a crucial role in sexual desire and maintenance of sexual activity (74), while the time shared between non-cohabiting partners, is the predominant predictor of negatively affected sexual interactions (75). Taking into consideration that many of the studies were conducted during complete quarantines and included participants who did not cohabit with their partner (36, 37, 58, 65), the impact of relationship status and a decrease in the frequency of partnered sexual interaction were anticipated. Surprisingly, this decrease applied for co-habiting partners in one of the studies as well (45). Though desire for sexual intercourse was reported as insignificantly changed or even higher by some of the studies, this did not progress into actual contact. This could be explained by findings which reported that the fear of contracting the virus minimized physical contact between partners (76). In addition, other findings supported that ruminating COVID-19-related conversations reduced the couples’ ability to avoid conflict, and decreased intimate expressions that could progress into sexual contact (77). The disagreement found between sexual desire and sexual intercourse, comes in accordance with what was found by Morokqff and Gillilland; males and females under the same stressful circumstances did demonstrate higher sexual desire, yet stressors prevented the progression of desire to actual sexual intercourse (78). Though sexual desire and frequency were expected to down escalate as age of participants increased (79), a portion of the studies did not verify the role of age in sexual life (37, 39, 45).

Likewise, the overall satisfaction deriving from sexual life was mostly affected in a negative fashion. Results showed that low sexual satisfaction was associated with health-related anxiety (33), something that has been documented; literature has highlighted the unfavorable relationship of anxiety and sexual contentment (80). Satisfaction deriving from sexual life is an integral part of sexual wellbeing and overall health (81). An important line of research has repeatedly shown that mentally healthy individuals are more satisfied by their intimate relationships, and vice versa; those with a more satisfying sex life exhibit a healthier mental state (80). However, the fact that some studies reported no change in satisfaction (49, 59) or other aspects of sexual wellbeing (48, 54) should not be neglected.

Based on the results, different aspects of sexual behaviors were found to have changed or to be newly added in individuals’ lives. A significant increase was noted with respect to masturbation (52, 55, 60, 61, 67) and pornography use (58, 61, 65, 66). An plausible explanation for this increase could be the fact that pornography has been found to be utilized as a stress coping method (82), or as a means to avoid emotional burden (83). However, the reliability of these findings should be considered carefully, as higher frequency of masturbatory practices appear more in males than females (84), and some of these studies have included solely male participants in their samples, while some samples constituted mostly by males. In addition, increases were reported with respect to online sexual activities such as cybersex, virtual dating, and creating and sharing sex-related digital content. Indeed, statistics on the topic has revealed the increase in dating applications’ downloads (85). Given that during the pandemic initiating new intimate relationships could be perceived as “unsafe,” virtual dating applications might offer a “safer” way to establish an alternative form of connection. It appears as intimacy quickly evolved and grew through online spaces, from emotional bonding *via* applications (86) to sex parties held *via* Zoom (87).

A wide heterogeneity was noted across studies. Each was conducted at different time points with respect to the severity of the pandemic. For example, a number of studies were conducted in countries with high number of infections and life losses, whereas in others -such as the example of Greece- studies were conducted when only a few cases of COVID-19 were being reported on a daily basis. Thereby, the impact of the pandemic could be characterized nothing but greatly variant. Thus, the implemented measures of social distancing were different when each study was conducted. For example in some European countries where the pandemic wave was milder restrictions were limited solely to the number of individuals that could gather, whereas in other countries complete ban of circulation was implemented. Another explanatory factor of the high heterogeneity could be the diverse samples between studies. Sampling varied significantly with respect to size, and demographic characteristics. Some included as small samples as of a few dozens of participants, while others recruited larger samples. In addition, both between and within studies sampling varied regarding gender, and relationship status; some included solely heterosexual or non-heterosexual participants, others included solely females or solely males, while others recruited mixed samples. The same issue occurred with respect to relationship status; some recruited exclusively married/cohabiting partners, while others solely single participants. The “when” and “how” is of great importance, as they could be the key factors in understanding the discrepancy of the findings.

### Strengths and limitations

Among the strengths of the present study is the systematic approach of the available data, including the search strategy, the selection process, as well as the extraction and presentation of information. The explicit eligibility criteria ensured the exclusion of misleading factors, such as established sexual or mental disorders prior the pandemic, while the data analysis assisted in the identification of trends between the included studies.

However, this review bears certain limitations. Though there was an effort to evaluate the impact of the stressful conditions formed by the pandemic on the sexual wellbeing and sexual behavior of individuals, specific factors limit the generalization of the findings. The fact that al included studies recruited convenient samples *via* online platforms constitute their findings vulnerable to selection and non-response biases, particularly regarding sexual behavior data. The meta-analysis was conducted on a small number of studies (especially for the male participants), which did not perform power calculation for their sample sizes, and thereby, their findings could be characterized as questionable. In addition, given the fact that a meta-regression could not be conducted, the mediating effect of other factors could not be determined.

## Conclusion

COVID-19 has forced circumstances such as limitation of the usual social connection and, in some cases, the sense of constant life threat (88), which mental health professionals should take into consideration when treating patients. They must be prepared to desensitize mental health patients regarding irrational fears deriving from the pandemic. In addition, the higher risk of mental health complication for individuals with pre-existing mental conditions should be under consideration (89). In relation to COVID-19 preventive measures and restrictions, sexual well-being seems to have been negatively influenced across several domains. As it appeared, the state of anxiety and stress could be considered as the key explanatory factor; those with experiencing stronger distress due to the restrictive measures seemed to have a less satisfying sexual life. Simultaneously, a rise in specific internet-based sexual behaviors such as pornography use, and cybersex were also prominent as alternative ways of sexual relief. Given that sexual health is an integral part of general health, this paper’s findings highlight that when the pandemic is surpassed and individuals begin to heal from this traumatic experience, surveillance and measurement of the final imprint on sexual wellbeing should be on the focus of clinicians and researchers.

## Data availability statement

The original contributions presented in this study are included in the article/supplementary material, further inquiries can be directed to the corresponding author.

## Author contributions

IM designed the study. IM and IK conducted the search and study selection and drawn the first draft of the manuscript. EK-M and KK extracted the data. IK performed the meta-analyses. GK and CP conducted the review and editing. All authors contributed to the article and approved the submitted version.
